# Tissue Specific Necroptosis in the Cervix, Uterus, and Placenta in an LPS-induced Preterm Birth Model

**DOI:** 10.1007/s43032-026-02062-2

**Published:** 2026-03-02

**Authors:** Sema Avcı, Mehmet Enes Sozen

**Affiliations:** Department of Histology and Embryology, Medical School, Alanya Alaaddin Keykubat University, Antalya, Turkey

**Keywords:** Preterm birth, Necroptosis, RIPK, MLKL, LPS

## Abstract

Preterm birth remains a preventable cause of death for both mothers and infants. However, due to its association with numerous biological mechanisms, its underlying processes remain poorly understood. Although components of the PANoptotic pathway have garnered increasing interest, particularly over the past decade, the literature concerning their role in preterm birth and pregnancy is still limited. To contribute to the understanding of mechanisms, we aimed to investigate the relationship between preterm birth and necroptosis markers such as receptor-interacting protein kinase-3 (RIPK3/RIP3) and mixed lineage kinase domain-like pseudokinase (MLKL) in the cervix, uterus, and placental tissues using lipopolysaccharide (LPS)-induced preterm birth model. Our objective was to determine the expression levels of RIPK3 and MLKL, explore their association with inflammation, assess how their levels change during preterm birth, and examine the potential role of Necrostatin-1 (NEC-1) in this mechanism. Our findings suggest that necroptosis is a component of LPS-mediated preterm birth, with RIPK3 and MLKL proteins being differentially expressed across the uterus, cervix, and placenta. The data indicate that necroptosis may be activated by distinct proteins depending on the reproductive tissue. Furthermore, the LPS-induced depletion of MLKL in the amniotic membrane suggests that this protein may play a key role in preterm membrane rupture, warranting further investigation. Although it is evident that the necroptosis pathway in preterm birth is a complex process which requires additional studies for complete elucidation, we believe that the results of our study will provide a foundation for future research in this area.

## Introduction

Historically, when we evaluate the cell death process, which is one of the important events in ensuring homeostasis, necrosis, and apoptosis appear as two critical factors. In the past, necrosis was considered an irregular cell death because of extensive cytotoxic effects caused by traditional molecular events, while apoptosis was defined as programmed cell death. Unlike necrosis, apoptosis is marked by nuclear condensation and fragmentation, the appearance of apoptotic bodies, and an intact plasma membrane [1]. While necrosis triggers an inflammatory reaction, apoptotic bodies are phagocytosed by macrophages, and inflammation does not occur [2]. Although necroptosis resembles necrosis in microscopic morphology, it is a type of apoptosis activated in a genetically regulated and programmed inflammatory environment [3, 4]. In necroptosis, fragmented chromatin, clustered cells, swollen cell bodies, organelles, and the disrupted cell membrane occur. Necroptosis, a recently identified form of programmed necrosis triggered by various extracellular and intracellular signals, involves the passive release of necroptotic cells from the compromised membrane into the extracellular matrix [1, 5]. Emerging literature underscores the potential contribution of regulated cell death pathways, including pyroptosis, apoptosis, necroptosis, and ferroptosis, in clearing infected or pre-cancerous cells and supporting the host immune response to pathogens [6].

The induction of necroptosis occurs in response to stimuli such as Toll-Like Receptor (TLR) agonists, ligands of death receptors, microbial pathogens, and receptor-mediated RIPK1 activation, with RIPK3 and its target MLKL playing pivotal roles in this pathway [7]. Inflammatory stimuli trigger the activation of intracellular adaptors Fas-associated death domain (FADD) and TNF-receptor-associated death domain (TRADD), which subsequently engage protein kinases. This activation brings together RIPK1, RIPK3, and MLKL, which are part of the necroptosis-inducing complex called the necrosome [3].

RIPK1 plays a dual role in regulating cell fate, inducing apoptosis and necroptosis in response to tumor necrosis factor (TNF), Fas, or LPS stimulation while promoting cell survival under certain conditions. When RIPK1 phosphorylation is impaired, it can associate with RIPK3 and caspase-8 to form complex II, facilitating both apoptosis and pyroptosis. In scenarios where caspase-8 activity is deficient, RIPK1 interacts with RIPK3 to form the necrosome, a complex that activates MLKL and triggers necroptosis. Additionally, under LPS stimulation, TLR4 engagement can lead to either cell survival or death through interactions involving RIPK1, RIPK3, and the adaptor protein TRIF [8]. RIPK1, which associates with the receptor, is pivotal in necroptotic cell death, potentially disrupting the oxidant-antioxidant balance and initiating a cytokine cascade [9]. The binding of tumor necrosis factor alpha (TNF-α) to tumor necrosis factor receptor 1 (TNFR1) induces the formation of complex I, where RIPK1 recruits the nuclear factor kappa B (NF-κB) essential modulator (NEMO; IKK-γ), thereby activating NF-κB and promoting inflammation. Following RIPK1 deubiquitination, complex I dissociates, leading to the assembly of complex IIa or IIb. Complex IIa contains FADD, caspase-8, and RIPK1, facilitating caspase-8 activation and apoptosis through caspase-3. If caspase-8 is inhibited, RIPK1 associates with RIPK3 to generate complex IIb [10]. Recent studies have shown that RIP3 can shuttle between the cytoplasm and the nucleus, whereas MLKL changes from a monomer to a dimer by RIP3-mediated phosphorylation. Under normal conditions, RIP3 stays bound to MLKL, keeping it inactive; however, MLKL undergoes significant conformational changes upon activation. Following phosphorylation by RIP3, MLKL oligomerizes and promotes the co-ubiquitination of tight junction proteins at the plasma membrane [11–13]. Considering the working principles of the pathway, RIPK3 (within complex IIb) and MLKL (outside complex IIb) [1] were selected as the proteins to be used in the study for the specific evaluation of necroptosis because they are not involved in the intermediate steps of apoptosis.

The cross-talk between pyroptosis, apoptosis, and necroptosis pathways is more pronounced in cellular responses to infection and is consistent with PANoptosis. PANoptosis is a cell death model in which pyroptosis, apoptosis, and necroptosis all occur within the same cell population. Understanding the basic mechanisms of various cell death pathways is crucial for developing new approaches against multiple diseases, including infections [14, 15]. Disruptions in necroptosis are associated with pathological conditions across various systems, including inflammatory diseases [16]. NEC-1, a necroptosis inhibitor, can effectively inhibit necrotic apoptosis, suppressing inflammation, endoplasmic reticulum stress, and the production of reactive oxygen species. NEC-1 treatment reduces the expression and phosphorylation of RIP1, RIP3, and MLKL, which is typically associated with decreased cell death, alleviated tissue damage, and better outcomes [17, 18]. However, the effects of NEC-1 on inflammation-mediated preterm birth are still unknown. In addition, the way the necroptosis pathway works in the preterm birth process is also unclear in the literature. Therefore, a more detailed study is needed to determine the role of necroptosis in the preterm birth process. When the data in the literature and previous studies are evaluated together, we hypothesize that "necroptosis has an active role in the infection-related preterm birth process." Our research aims to determine the effectiveness of necroptosis in the cervix, uterus, and placenta during preterm birth, the role of RIPK3 and MLKL proteins belonging to this pathway, and to reveal how NEC-1 application changes the localization and amount of proteins in the listed tissues.

## Materials and Methods

### Animal Groups

CD-1 female mice in the estrus phase of eight to twelve weeks were placed in individual cages with males of the same breed for mating, and copulation plugs were checked the next day (= 0.5th day). The main groups in the experimental model were as follows: Non-Pregnant Control group (NPC), Pregnant Control group (PC), Dimethyl Sulfoxide Intraperitoneal (IP) application group (DMSO), Necrostatin-1 IP application group (NEC-1), Lipopolysaccharide IP application group (LPS), and the combined NEC-1 and LPS IP application group (L + N). Each group consisted of 6 subjects (*n* = 6). Group-specific procedures were carried out on gestational day 14.5 in CD-1 mice, whose term gestational length is 18–19 days [19]. 100 μl of the agent was injected IP according to the group in which the subject was located. After the DMSO, LPS, and NEC-1 were applied to CD-1 mouse groups at 14.5 dpc (days post coitus).

Pregnant subjects were excluded from the experiment if their average weight (39.5 gr) was outside of ± 25%. This situation, which may indicate an increase in fat and circulatory problems, was implemented because it aimed at all subjects in similar conditions. In addition, since the absence of a pup descending to the cervix after LPS induction was associated with insufficient functioning of the LPS or application problems, the subject was excluded from the experiment, and a new one was planned to be added instead. However, no addition was needed during the experiment. The localization and protein expression levels of RIPK3 and MLKL proteins, which are components of necroptosis, in the cervix, uterus and placenta tissues of the subjects were evaluated immunohistochemically and by western blot method.

### Study Design

To induce preterm labor in mice, LPS (E.coli-Lipopolysaccharide-Escherichia coli O111:B4; L2630-10 mg, obtained from Sigma bacterial wall) was administered IP in two equal doses totaling 50 µg/kg, given at 3-h intervals. NEC-1 (480065, Merck), 25 mM (0.7 mg NEC-1/100 μl 5% DMSO-D2650, Sigma) was administered in two doses at 4:00 and 7:00 p.m. Studies have demonstrated an inhibitory effect at a dose of 20 mM for NEC-1 [20]. A significant limitation of NEC-1 is its short half-life of approximately 1–2 h in rats (1.8 ± 0.9 h for intravenous and 1.2 ± 0.3 h for oral administration) [21]. In the group receiving both LPS and NEC-1 (L + N), NEC-1 was administered IP 1 h before LPS injection. Water and food intake was ad libitum. Subjects were kept in a 12-h day/night and 22 (± 2) ^0^C constant temperature cycle.

The primary outcome measure was preterm birth (at least one or more pups seen in the cage or lower vagina within 24 h). The standard observation period was terminated at 14:00 on day 15.5 after the treatments, and all subjects were sacrificed. Just before euthanasia, the mice were anesthetized with 0.017 ml/kg Avertin (2.5% tribromoethanol and 2.5% tertiamyl alcohol), and cervical dislocation was performed. Tissues were then collected for experiments.

### Immunohistochemical Staining Protocol

After the tissues were removed, 10% formalin fixative (818708; Merck) was prepared, and the cervix, uterus, and placenta tissues were kept in the solution for 24 h. After incubation, the tissues were washed with tap water for 3 h and kept in 70%, 80%, and 90% ethanol (459844; Sigma-Aldrich) series for 24 h, respectively. After being kept in 100% ethanol for 3 h, the tissues were made transparent in xylenes (534056; Sigma-Aldrich) for approximately 5 min and then embedded in paraffin. The tissue blocks were cut into 5 µm thick. 1X PBS (P4417-100TAB; Sigma-Aldrich) solution was used to wash the sections, and then the sections were treated with a citric acid (100244; Merck) buffer before staining. Blocking was achieved with Ultra V Block (TA-125-UB Thermo Scientific) using 3% H2O2 Solution (106009; Merck, 18312; Sigma) to eliminate endogenous peroxidase activity. The following markers, which are explained in the main text and thought to have a role in preterm birth, were used to evaluate the protein localization and expression levels of the tissues to create a descriptive map. As primary antibodies, RIPK3 (Thermo, PA5-19956, dilution:1/100), MLKL (Thermo, MA5-31971, dilution:1/100), and Goat Anti-Rabbit (Vector, BA-1000, dilution:1/500) were used as secondary antibodies. After applying a diaminobenzidine tablet (D4168; Sigma) as chromogen and Mayer's hemalum solution (109249, Merck) for contrast staining, the imaging phase was started. The immunostained sections were examined under a Zeiss Axioplan microscope (Germany), and images were captured with a Nikon MQD 42070 (4 K) camera system (Japan).

### Histological H-score Evaluation

The immunohistochemical staining intensity of the epithelium was evaluated using the H-Score for the markers investigated in each experimental group. For the cervix, both endocervical and ectocervical epithelium were examined; for the uterus, the uterine epithelium; and for the placenta, the amniotic epithelium, junctional zone (JZ) and labyrinth zone (LZ) were analyzed. In the H-Score analysis, regions were assigned numerical values ranging from 0 to 3 based on staining intensity (-/ + : Negative, + : Mild, +  + : Moderate, +  +  + : Strong). The average values obtained for each tissue were then presented in a table.

### Evaluation of Histological Staining Intensity

Immunohistochemical staining intensity was measured in three different images (40X) for three randomly selected subjects in each group using the Image J (1.52 R, National Institutes of Health, USA) Fiji supplement, and the staining intensity obtained after subtracting the background staining area from the DAB stained area was using the GraphPad Prism v9 program to evaluate whether the difference between the groups (*p* < 0.05) was significant.

### Protein Extraction and Western Blot

To prepare cervix, uterus, and placenta tissue lysates, lysis buffer solution (Tris, 108387; Merck) and protease inhibitor cocktail (P8340; Sigma) were used, and the amount of protein in the lysates was measured with the BCA protein assay kit (23225; Pierce). SDS–Polyacrylamide gel electrophoresis was performed by loading 50 ug of protein for each well. For this procedure, electrophoresis was performed using 4–12% Bolt Bis Tris Plus Gel system (NW04120BOX; Invitrogen and 4–20% TGX Mini Protean Gel (4568094; BioRad) at 120 V for 90 min. Then, after blotting with the BioRad Gel Turbo TransBlot system (1704150; BioRad), the membranes were blocked using 5% nonfat dry milk powder (Blotting Grade Blocker, 1706404; Bio-Rad) for 1 h at room temperature. The imaging step was started after incubating the membranes with primary antibodies on a shaker at + 4 °C overnight. The primary antibodies are as indicated below.

RIPK3 (Thermo, PA5-19956) and MLKL (Thermo, MA5-31971) were used as primary antibodies. Goat Anti-Rabbit was used as the secondary antibody (Abcam; ab6721).

After incubation with primers, the membranes were washed with Tris-buffered saline-TBS-T (1% Tween-20, 822184; Merck) and incubated with secondary antibodies for 45 min at room temperature. The membranes were washed with TBS-T and incubated with Super Signal Chemiluminescence (CL)-HRP (A45918; Thermo Scientific) and visualized with Azure Imaging Biosystem (Color C 600).

### Statistical Analyses

For Western blot analyses, protein and beta-actin band measurements were performed using the Image J (1.52 R, National Institutes of Health, USA) program. Then, protein/beta-actin values ​​were statistically evaluated using the GraphPad Prism v9 program. The normality of the obtained data was assessed using the Kolmogrov-Smirnov test, and the homogeneity was evaluated using the Brown-Forsythe test. For parametric data obtained with numerical measurements, the significance of the difference between the groups was examined using the one-way analysis of variance (ANOVA) test. In cases where the difference between the groups was significant, the Tukey multiple comparison test was used after the ANOVA test. The statistical significance limit will be accepted as *p* < 0.05. Parametric data were used as mean ± standard deviation; for non-parametric data, median and 25–75 percentile values ​​were used to determine the difference. The difference between the groups was marked using asterisks appropriate to the power of the difference in the group bars.

## Results

The onset of bleeding and birth was delayed in the LPS (L) + NEC-1 (N) group compared with the LPS-only group. In the LPS-treated group, bleeding or birth occurred in all subjects by 07:00. However, in the L + N group, the onset was delayed by at least 2 h in 4 out of 6 subjects. Although the pups were observed descending into the vagina, the time required for complete uterine emptying was prolonged. In this group, bleeding or birth occurred within the observation period, which lasted until 14:00. The LPS-only group was euthanized immediately after the onset of labor or bleeding, while all other pregnancy groups were monitored until 14:00 and then euthanized.

The variability observed in the L + N group may be related to differences in litter size and, consequently, the dose or distribution of NEC-1. Alternatively, these findings may suggest that NEC-1 mitigates inflammation by inhibiting necroptosis, thereby delaying the onset of preterm labor. Notably, placentas in the L + N group appeared fresher and more similar to those of the control group on macroscopic examination compared with the LPS group. Fetal and placental weights were not assessed in this study.

### RIPK3 Expressed in the Cervix and Uterine Epithelium was Immunohistochemically Increased in the LPS Group and Decreased in the L + N Group Compared to the LPS Group

**Fig. 1:**
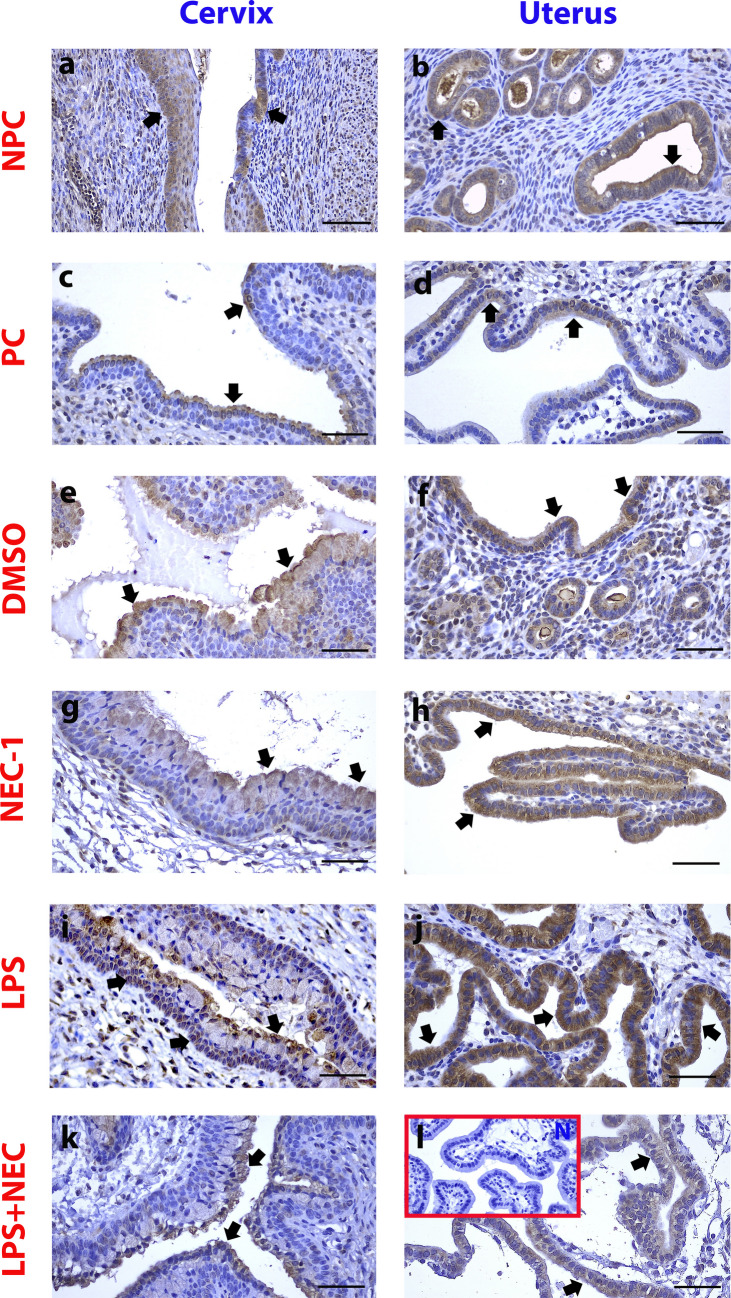
RIPK3 expressed in the cervix and uterine epithelium was immunohistochemically increased in the LPS group and decreased in the L + N group compared to the LPS group. NPC: **a**,**b**: Non-Pregnant Control, PC: **c**,**d**: Pregnant Control, DMSO **e**,**f**: Solvent control, NEC-1 **g**,**h**: Necrostatin-1(25 mM), LPS **i**,**j**: Lipopolysaccharide, 25 µg, L + N **k**,**l**: Lipopolysaccharide 25 µg + Necrostatin-1 (25 mM). Black Arrowheads: Positively stained cells, N: Negative Control, Scale bar: 50 µm

In the cervix, in the control groups (NPC, PC, DMSO, NEC-1), in the ectocervical area epithelium (Fig. 1-a,c,e,g/black arrows) and the uterine epithelium in the control groups (Fig. 1-b,d,f,h/black arrows), cytoplasmically positive stained cells were more intensely stained in the LPS group in the cervix and uterine epithelium (Fig. 1-i,j/black arrows). The staining intensity differed significantly from the control groups (*p* < 0.05). In the L + N group, it was observed that NEC-1 application suppressed the effect of LPS in both tissues, creating a response similar to the control (*p* < 0.05) (Fig. 1-k,l/black arrows) (Fig. 5-a,c, and i).

#### MLKL Expressed in the Cervix and Uterine Epithelium Increased Immunohistochemically in the LPS Group and Decreased in the L + N Group Compared to the LPS Group

**Fig. 2:**
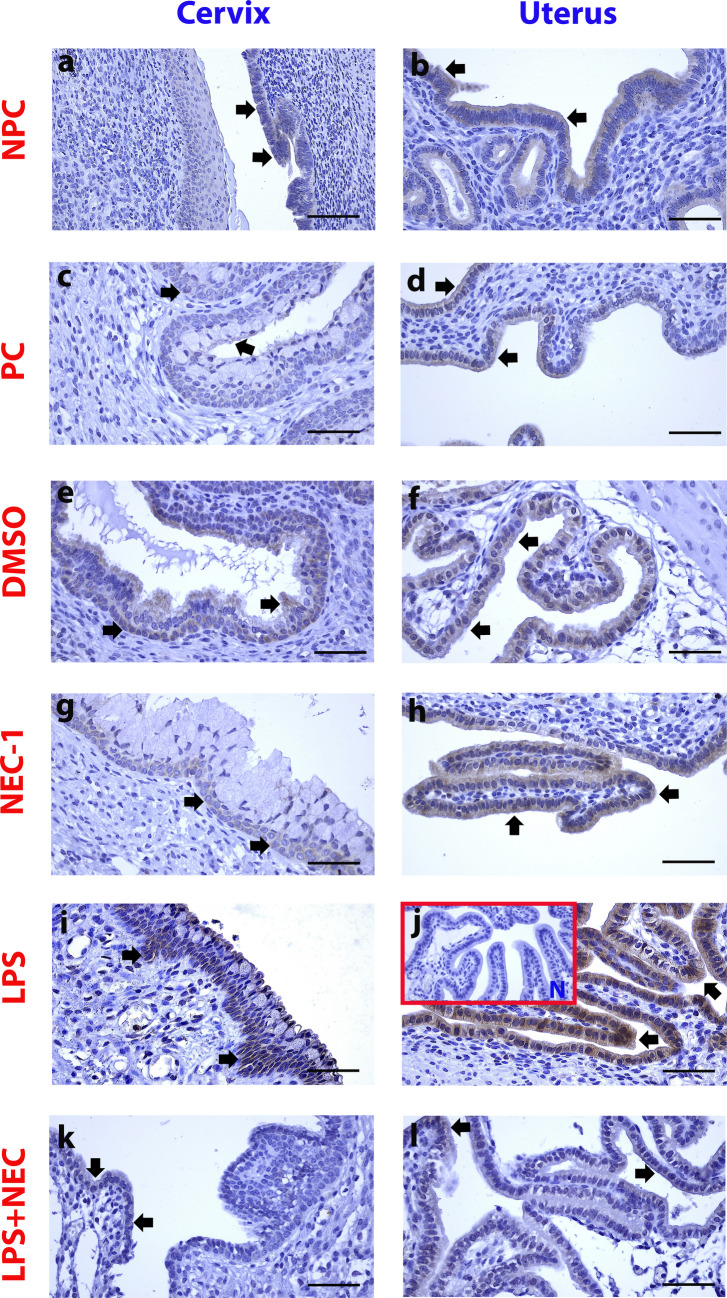
MLKL expressed in the cervix and uterine epithelium increased immunohistochemically in the LPS group and decreased in the L + N group compared to the LPS group. NPC: **a**,**b**: Non-Pregnant Control, PC: **c**,**d**: Pregnant Control, DMSO **e**,**f**: Solvent control, NEC-1 **g**,**h**: Necrostatin-1 (25 mM), LPS **i**,**j**: Lipopolysaccharide 25 µg, L + N **k**,**l**: Lipopolysaccharide 25 µg + Necrostatin-1 (25 mM). Black Arrowheads: Positively stained cells, N: Negative Control, Scale bar: 50 µm

MLKL protein was positively stained in the cervix and uterine epithelium in the control groups (Fig. 2-a-h/black arrows). The staining intensity was more higher in the epithelial area in the LPS group compared to the control groups in both tissues (Fig. 2-i,j/black arrows) (Fig. 5-b,d and i) (*p* < 0.05). In contrast to the group treated with LPS alone, the L + N group demonstrated a reduction in expression within the cervix and uterine epithelium (Fig. 2-k,l/black arrows) (Fig. 5-b,d and i) (*p* < 0.05).

#### Immunohistochemical Expression of RIPK3 in Placenta JZ, DZ, and A Was Similar in all Groups

**Fig. 3:**
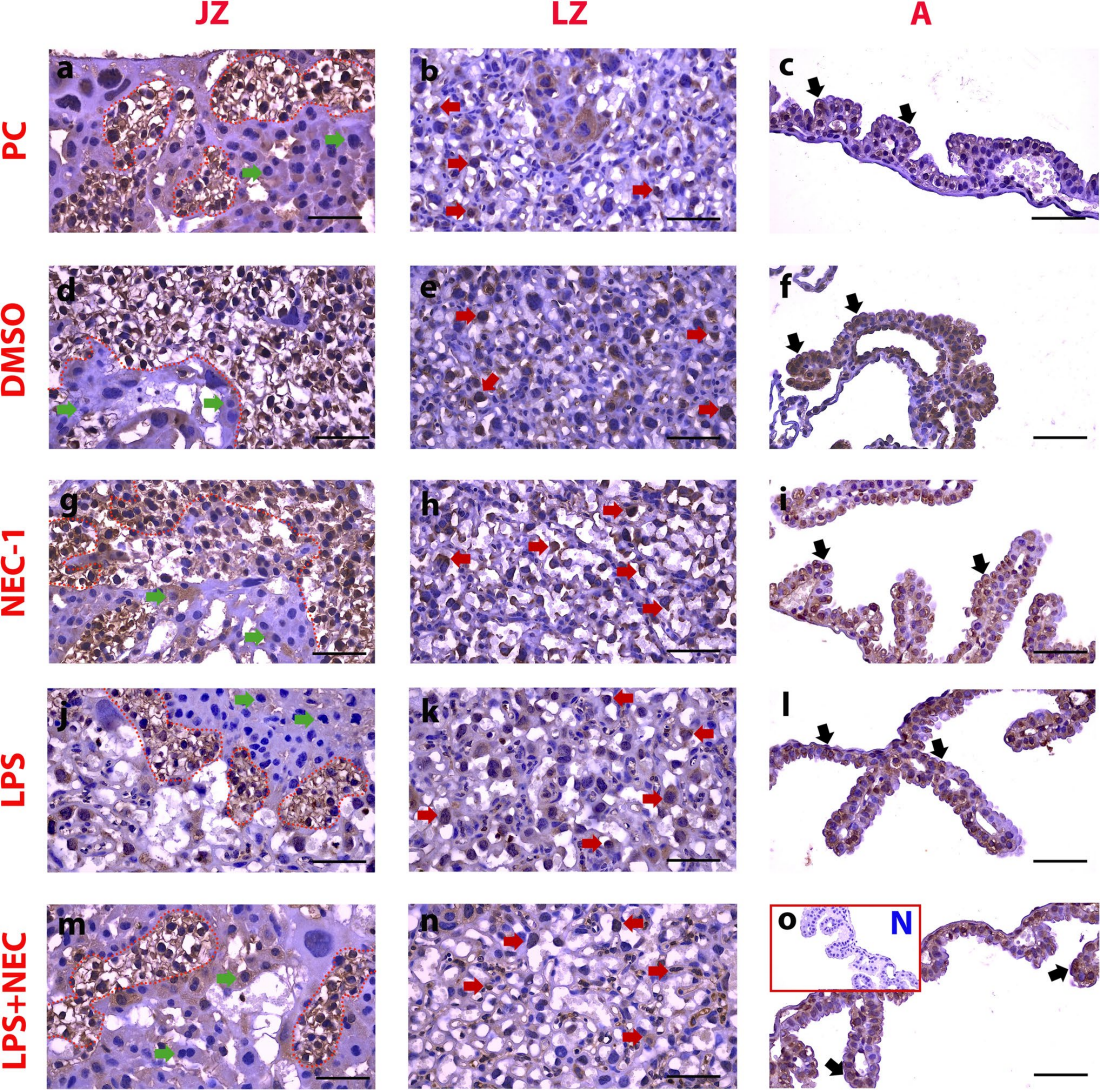
Immunohistochemical expression of RIPK3 in placenta JZ, DZ, and A was similar in all groups. PC: **a**,**b**,**c**: Pregnant Control, DMSO: **d**,**e**,**f**: Solvent control, NEC-1 **g**,**h**,**i**: Necrostatin-1 (25 mM), LPS **j**,**k**,**l**: Lipopolysaccharide 25 µg, L + N **m**,**n**,**o**: Lipopolysaccharide 25 µg + Necrostatin-1 (25 mM). JZ: Connection Zone, LZ: Labyrinth Zone, A: Amniotic Membrane. Black Arrowheads: Positive staining cells, Green Arrowheads: Spongiotrophoblast, Red Arrowheads: Trophoblast, Red Dashed Line: Glycogen islands, N: Negative Control, Scale bar: 50 µm

The placental junction zone (JZ), labyrinth zone (LZ), and amniotic epithelium (A) were evaluated regionally due to differences in staining patterns. Positive staining areas of RIPK3 were in glycogen islands in all groups in JZ (Fig. 3-a,d,g,j,m/red dashed line), while it was very weak in spongiotrophoblast cytoplasms (Fig. 3-a,d,g,j,m/green arrowheads). Trophoblast cytoplasms were positively stained in LZ (Fig. 3-b,e,h,k,n/red arrowheads). When the groups were compared, there was no significant difference between them (Fig. 5-e,i) (*p* > 0.05). Cell cytoplasms in the amniotic membrane epithelium were positively stained (Fig. 3-c,f,i,l,o/black arrowheads). No statistically significant difference was observed between the groups (Fig. 5-i,g) (*p* > 0.05).

#### Immunohistochemical Expression of MLKL in JZ, LZ, and A of the Placenta Was Decreased in the LPS Group

**Fig. 4:**
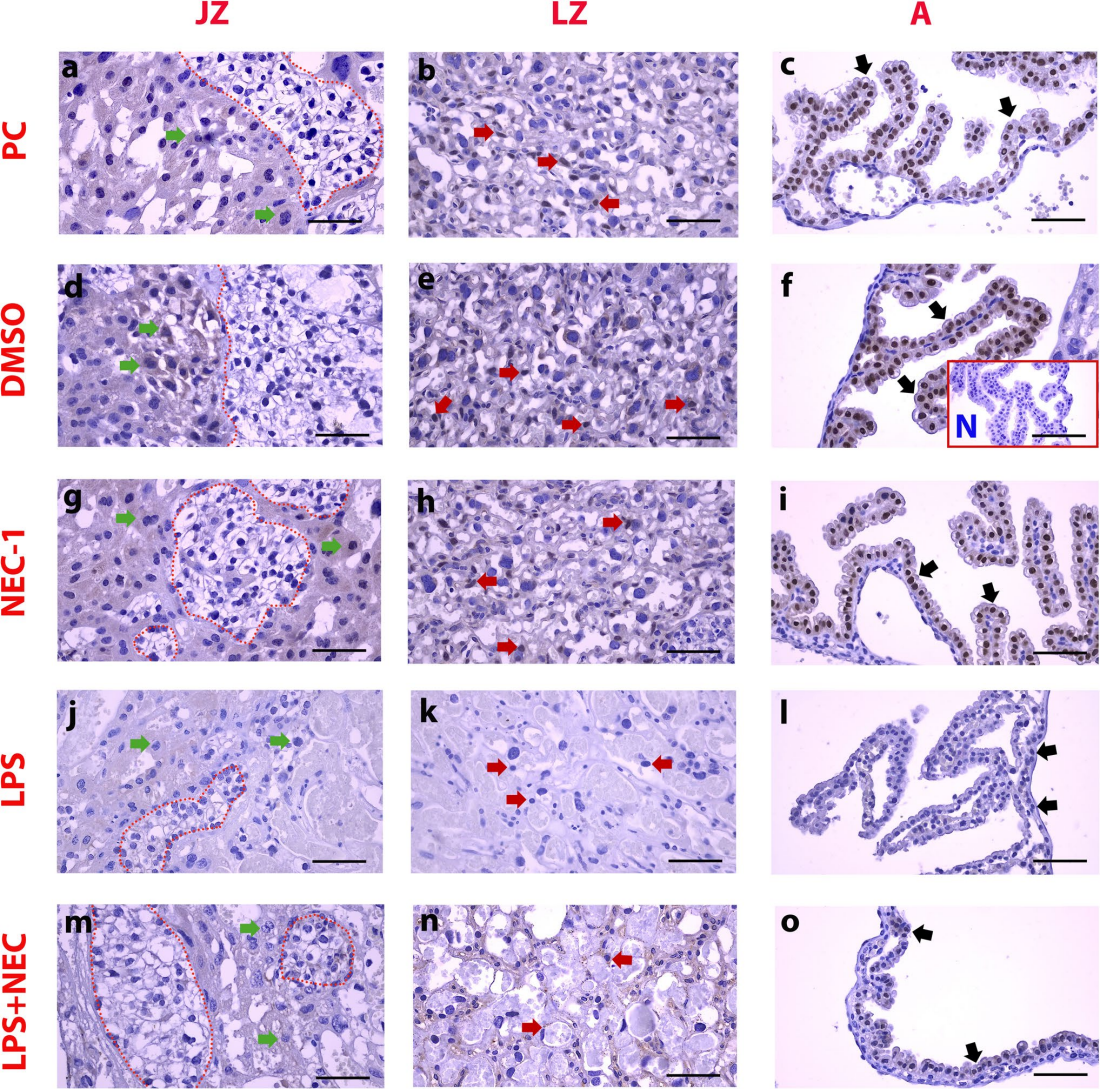
Immunohistochemical expression of MLKL in JZ, LZ, and A of the placenta was decreased in the LPS group. PC: **a**,**b**,**c**: Pregnant Control, DMSO: **d**,**e**,**f**: Solvent control, NEC-1 **g**,**h**,**i**: Necrostatin-1 (25 mM), LPS **j**,**k**,**l**: Lipopolysaccharide 25 µg, L + N **m**,**n**,**o**: Lipopolysaccharide 25 µg + Necrostatin-1 (25 mM). JZ: Junction Zone, LZ: Labyrinth Zone, A: Amniotic Membrane. Black Arrowheads: Positive staining cells, Green Arrowheads: Spongiotrophoblast, Red Arrowheads: Trophoblast, Red Dashed Line: Glycogen islands, N: Negative Control, Scale bar: 50 µm

**Fig. 5:**
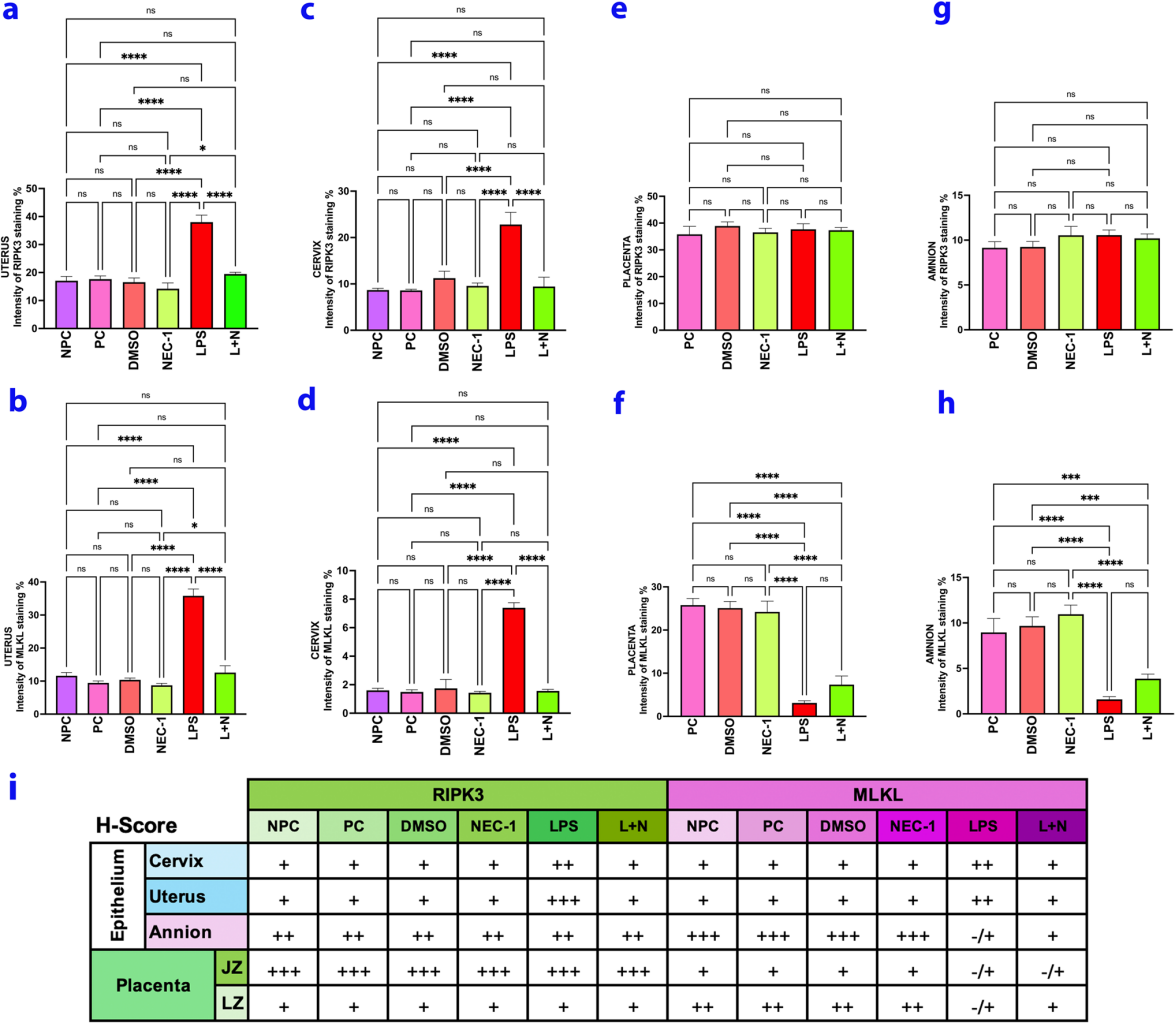
RIPK3 and MLKL Staining Intensity and H-Score (i) Analysis in Uterus **a**,**b**, Cervix **c**,**d**, Placenta **e**,**f** and Amniotic Membrane **g**,**h**. NPC: Non-Pregnant Control, PC: Pregnant Control, DMSO: Solvent control, NEC-1: Necrostatin-1 (25 mM), LPS: Lipopolysaccharide 25 ug, L + N: Lipopolysaccharide 25 µg + Necrostatin-1 (25 mM)

The placental staining pattern was different for MLKL protein than RIPK3. Although weak staining was observed cytoplasmically in spongiotrophoblasts (Fig. 4-a,d,g,j,m/green arrowheads), staining was negative in glycogen islets (Fig. 3-a,d,g,j,m/red dashed line). Staining was positive in trophoblasts in LZ (Fig. 4-b,e,h/red arrowheads). In addition, expression strength was significantly decreased in the LPS group (Fig. 4-j,k) compared to control groups (Fig. 4-a,b,d,e,g,h) (Fig. 4-j,k) (Fig. 5-f,i) (*p* < 0.05). Although an increase was observed in LZ compared to LPS in the L + N group (Fig. 4-m,n/red arrowheads), it was not significant (Fig. 5-f,i) (*p* > 0.05).

Perhaps the most striking difference is in the amniotic membrane for MLKL expression. While the amniotic membrane structure specifically expresses MLKL protein nuclearly (Fig. 4-c,f,i/black arrowheads), the expression strength in the amniotic membrane decreased significantly in the LPS group (Fig. 4-l/black arrowheads). Although weak nuclear expression was observed in the L + N group (Fig. 4-o/black arrowheads), the expression strength did not show a significant increase compared to LPS (Fig. 5-h,i) (*p* > 0.05).

#### RIPK3 Increased in the LPS Group and Decreased in the L + N Group in the Uterus and Cervix, and RIPK3 Did not Change in the Placenta

**Fig. 6:**
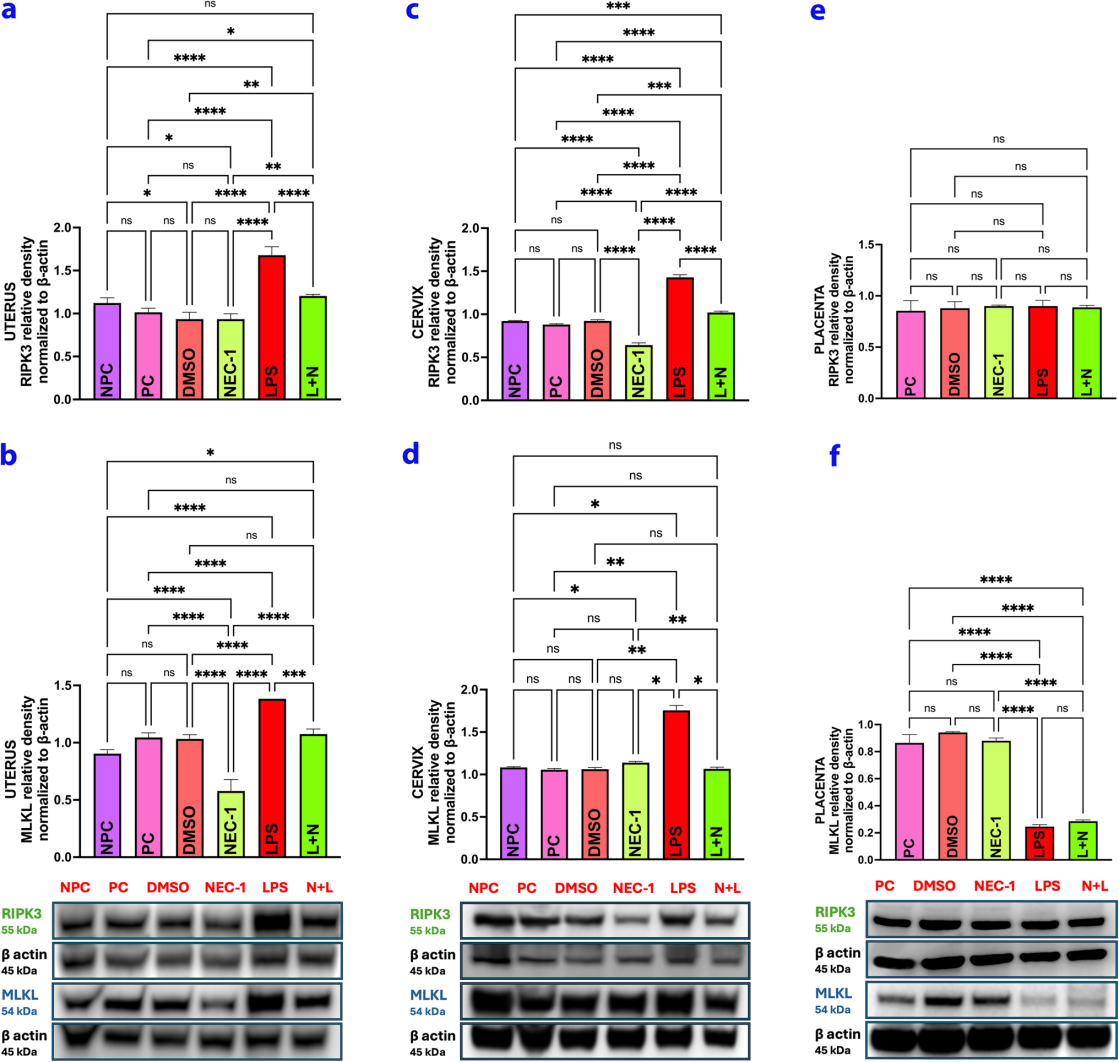
Western Blot Analysis Graphs of Uterus **a**,**b**, Cervix **c**,**d**, Placenta **e**,**f** Tissues. RIPK3 and MLKL increased in the LPS group in the uterus and cervix while decreased in the L + N group. While RIPK3 did not change in all groups in the placenta, MLKL decreased in the LPS and L + N groups. NPC: Non-Pregnant Control, PC: Pregnant Control, DMSO: Solvent control, NEC-1: Necrostatin-1 (25 mM), LPS: Lipopolysaccharide 25 µg, L + N: Lipopolysaccharide 25 µg + Necrostatin-1 (25 mM)

RIPK3 protein levels in the cervix, uterus, and placenta were similar in the control groups except in the NEC-1 group (Fig. 6-a,c,e) (*p* > 0.05). RIPK3 protein levels in the cervix in the NEC-1 group were lower compared to the other groups, and the difference was significant (Fig. 6-c) (*p* < 0.05). While expression did not change in the placenta in the LPS group (Fig. 6-e) (*p* > 0.05), it increased in the cervix and uterus (*p* < 0.05), and the decrease in protein levels in these tissues was significant in the L + N group compared to LPS (Fig. 6-a,c) (*p* < 0.05).

#### MLKL Expression Increased in the Uterus and Cervix but Decreased in the Placenta in the LPS Group, whereas it Was Reduced in all Tissues in the L + N Group

When the MLKL protein levels in the cervix, uterus, and placenta were compared with the control groups, they were similar to each other except for the NEC-1 group in the uterus (Fig. 6-b,d,f) (*p* > 0.05). In the NEC-1 group, the amount of MLKL protein in the uterus was significantly decreased compared to the control groups (Fig. 6-b) (*p* < 0.05). An increase in protein in the cervix and uterus was observed after LPS application. The decrease seen in the L + N group was significant when compared to LPS (Fig. 6-b,d) (*p* < 0.05). In the placenta, there was a significant decrease in the LPS and L + N groups compared to the control groups (Fig. 6-f) (*p* < 0.05).

## Discussion

Preterm birth is most frequently associated with infection in the literature. An increase in vaginal pathogen colonization is considered the primary possible mechanism behind infection-associated preterm birth [22] and LPS is frequently used in experimental animal models that mimic infection-mediated preterm birth [23]. Likewise, the placenta consists of multiple zones and cell populations, which can generate different responses [24]. Therefore, in evaluating protein localizations and cellular expression statuses, different markers were used to assess the status of cells, placental zones, and changes in the cervix and uterine tissues. Even though fetal placental membranes offer physical protection against LPS and microbial agents [25], it has been shown in the literature that vaginal pathogens can reach the amniotic membrane [26].

The placenta, a vital maternal–fetal interface, plays a key role in numerous physiological functions such as bidirectional molecular transport, endocrine regulation, immune defense, and maintaining maternal–fetal tolerance. Necroptosis has been identified to have a dual function within placental biology. While necroptotic signaling is essential for proper placental development and fetal organ formation, its dysregulation is implicated in the development of pregnancy-related disorders. Recent studies have associated abnormal necroptosis with the pathogenesis of significant pregnancy complications, including preeclampsia, fetal growth restriction, recurrent miscarriage, and gestational diabetes mellitus [27]. In our study, no significant difference was observed in RIPK3 expression and protein levels in the placenta, while NEC-1 application did not cause an effect on RIPK3. However, MLKL expression and protein levels decreased in the placenta after LPS application. Although slight expression was observed in LZ in the placenta after NEC-1 application, no significant increase was observed. NEC-1 could not reverse the effect of LPS at the protein level in the L + N group. The results may suggest that LPS directly suppresses MLKL and prevents necroptosis. In addition, although not mediated by RIPK3, the fact that MLKL did not increase after LPS application in the placenta suggests that a different apoptotic or necroptotic pathway other than canonical necroptosis may be activated. Namely;

Although NEC-1 is used to determine necroptosis and target RIP1 kinase activity in a wide variety of pathological cell death events [18], not only does it inhibit RIP1, but it also diminishes RIP3 expression and phosphorylation, thereby hindering the formation of the RIP1-RIP3 complex by attenuating their interaction, as shown in many disease contexts [18, 28]. In addition, the literature has reported that NEC-1 inhibits necroptosis and is associated with apoptosis inhibition [29]. In scenarios where apoptosis is mediated through RIP1, NEC-1 can inhibit RIPK1 activity and complex IIa formation, thus blocking cell death. However, if apoptosis occurs independently of RIP1, NEC-1 has no effect. Another viewpoint proposes that inhibition of necroptosis by NEC-1 may lead to a shift toward apoptotic cell death [18, 30]. Therefore, the fact that NEC-1 does not have an inhibitory effect on RIPK3 in the placenta suggests that the axis has shifted to an apoptosis-independent direction from NEC-1 and that the protein level may have decreased due to the disruption of the necrosome structure and insufficient phosphorylation of MLKL due to inadequate formation of the RIP-RIP3 complex.

Our literature defines premature rupture of membranes (PROM) as the tearing of fetal membranes before birth contractions begin. This event can lead to spontaneous labor but also increases the risk of intra-amniotic infection and placental abruption. The fetal membranes are composed of two layers: the inner side amnion and the outer side chorion, which is attached to the decidua of the endometrium. As a result, the amnion is sensitive to alterations in the amniotic cavity, while the chorion is crucial for sustaining immune tolerance at the maternal–fetal boundary. Selective apoptosis drives membrane remodeling toward the end of pregnancy, leading to membrane weakening. Inflammatory processes can activate proteases within the fetal membranes, compromising their integrity and increasing susceptibility to rupture. While intrauterine infection frequently accompanies membrane rupture, it remains uncertain whether infection is a cause or consequence of the rupture [31–33].

As a remarkable result, the depletion of nuclear MLKL expression observed immunohistochemically in the amniotic membrane in the LPS-treated group suggested that MLKL may be a protein associated with membrane rupture observed in preterm birth. Phosphorylation of MLKL is associated with ATP depletion and plasma membrane disruption. It can be thought that MLKL, which carries out membrane translocation after oligomerization following RIPK3-mediated phosphorylation, is depleted by disrupting membrane integrity [27] and aggravating the inflammatory responses of surrounding cells [34]. However, this process, which is related to the amniotic membrane, needs to be investigated more.

Necroptosis is involved in the pathogenesis of bacterial infections. Limiting these infections and generating a balanced response is vital for survival [35]. At the right time, the destruction of the epithelium, which acts as a barrier for bacteria, can facilitate bacterial progression [36]. Necroptosis is a possible route to overcome the epithelial barrier. In some cases, necroptosis can limit excessive infection [37]. The dual effect of necroptosis may be related to the type of bacteria and the severity of the infection, and more studies are needed to determine how the balance is achieved in cases where we can talk about a beneficial or harmful effect [38].

LPS activates necroptosis signaling and enhances the expression of RIPK1, RIPK3, and MLKL, critical mediators of programmed necrotic cell death [39]. In the literature, changes in necroptosis components in different tissues have been evaluated after LPS induction, and some of them have reviewed the effect of NEC-1 on necroptosis proteins. However, no study has been found in the literature evaluating the relationship between necroptosis and preterm birth within the framework of reproductive tissues. When we examine some of these studies that are similar to our research in terms of methodology. In a study evaluating necroptosis components in the hypothalamic axis, 1 mg/kg NEC-1 dissolved in 2% DMSO was administered IP 30 min before the IP injection of LPS, and the subjects were sacrificed 4 h after the injection of LPS (100 μg/kg/IP). NEC-1 has been shown to suppress necroptosis components [40].

In a different study, 1 mg/kg/ip NEC-1 in 5% DMSO was administered to mice together with LPS. When LPS was administered together with a caspase inhibitor, phospho-MLKL (p-MLKL) increased, and a transient interaction was observed between extracellular cold-inducible RNA-binding protein (eCIRP) and MLKL, and eCIRP could exit the cell through pores formed by p-MLKL. NEC-1 suppressed the increase. Within this framework, necroptosis is believed to be a regulated death pathway dependent on MLKL activation, leading to DAMP release. eCIRP, released during necroptosis, further intensifies the septic inflammatory response [41]. In another study, in the sepsis model associated with lung infection, NEC-1 was administered at 20 mg/kg/iv 10 min before the model was established. As a result, MLKL-NLRP3-mediated necroinflammation was significantly upregulated in the lung tissue of septic mice, and this situation could be attenuated by the specific inhibitor NEC-1 [42]. Increased RIPK1, RIPK3, and MLKL activity in LPS-induced thermal hyperalgesia were reversed by NEC-1 [43]. Again, LPS increased p-RIPK1, p-RIPK3, and p-MLKL in acute lung injury, and the necroptotic effect was reversed by NEC-1 [44].

RIPK1 and RIPK3 have been implicated in the development of renal fibrosis after ischemia–reperfusion and kidney injury. NEC-1-treated mice showed reduced kidney damage at 24 h compared to controls [45]. Necrostatin-1 also had a positive effect on cognitive decline by reducing the increases in protein levels of RIPK1, RIPK3, MLKL, and p-MLKL associated with necroptosis in the hippocampal tissue of paclitaxel-treated mice [46]. In another study, It was observed that necroptosis contributed to the formation of damage and increased total and phosphorylated RIP3 and MLKL proteins in deoxynivalenol-induced liver injury. NEC-1 administration inhibited necroptosis and reduced damage [47]. In a different research, it was shown that the expression of RIPK1, RIPK3, and p-MLKL increased in preeclampsia, while pre-treatment with NEC-1 decreased the expression [48].

**Fig. 7:**
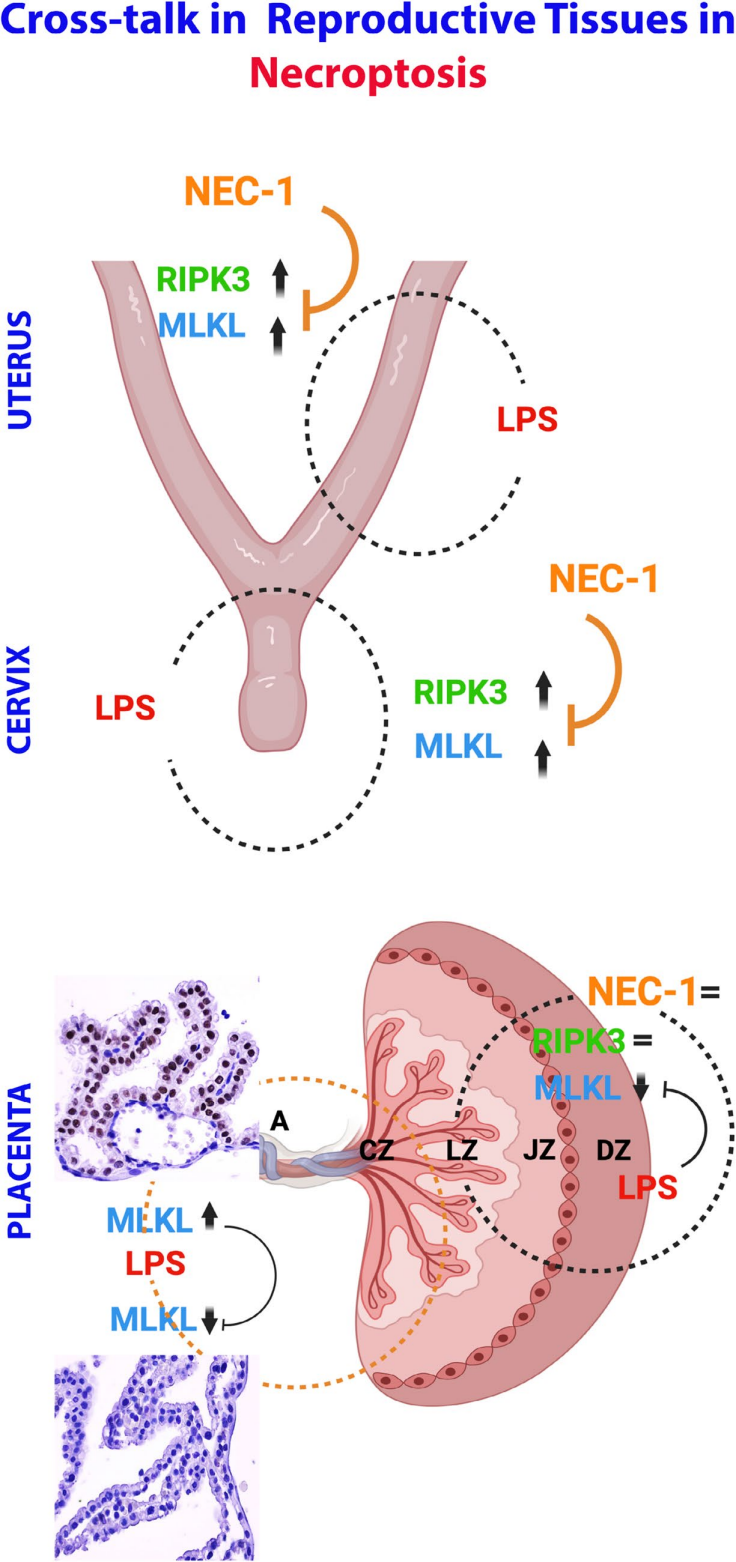
Possible summary of reproductive tissue cross-talk in necroptosis. LPS application upregulates RIPK3 and MLKL proteins in the uterus and cervix, while NEC-1 downregulates these proteins via MLKL in the uterus and RIPK3 in the cervix. LPS application did not change RIPK3 in the placenta but suppressed MLKL. In the amniotic membrane, LPS application depleted MLKL

As seen in the studies mentioned above, RIPK3 and MLKL proteins are upregulated after LPS, and this increase is suppressed after different doses of NEC-1 pretreatment. Our research showed that RIPK3 and MLKL expression increased in the uterine epithelium in the LPS-applied group, and a similar upregulation was observed at the protein level. A parallel increase was also present in the cervix. These increases were significant and, similar to the literature, were suppressed after NEC-1 pretreatment. However, another piece of information that we will add to the literature is that NEC-1 in the uterus may suppress necroptosis activated by the increase in RIPK3 and MLKL after LPS application, probably via MLKL, and in the cervix via RIPK3. Based on the NEC-1 effect seen at the protein level, it has been suggested that NEC-1 does not suppress RIPK3 in the uterus. Thus, the decrease seen in the L + N group is probably achieved by restricting MLKL phosphorylation via RIP1. In the cervix, NEC-1 appears to control necroptosis by specifically suppressing RIPK3. This pathway suggests that a cascade occurs, resulting in the suppression of RIP1 and, therefore, RIPK3 by reducing ROS or the suppression of MLKL and thus limiting necroptosis by reducing RIPK3 conversion directly via RIP1, based on the multifaceted mechanism of action of NEC-1 [18]. This pathway may also inhibit phospho-RIP1-dependent apoptosis and induce caspase 8-dependent apoptosis. In summary, NEC-1 administration in LPS-induced preterm birth suppresses necroptosis at varying levels in reproductive tissues. The hypothesized interactions in tissues are summarized in Fig. 7.

## Conclusion

Our study presents findings that may contribute both to clinical treatment perspectives and to future research efforts in this area. Notably, our findings suggest that necroptosis is a component of LPS-induced preterm birth and that the expression of necroptosis-related proteins differs across reproductive tissues, including the uterus, cervix, and placenta. Moreover, the necroptosis inhibitor NEC-1 appears to exert tissue-specific effects by selectively targeting different proteins. Additionally, the depletion of MLKL in the amniotic membrane following LPS exposure raises the question of whether this protein plays a key role in the premature rupture of membranes, warranting further investigation. While it is evident that the necroptosis pathway in preterm birth represents a complex process that requires further elucidation, we believe that our findings will contribute valuable new data to the existing literature and encourage the design of future studies. Determining the status of necroptosis markers in different tissues and conditions may provide novel diagnostic molecules from a clinical perspective, while targeted therapies may help mitigate adverse outcomes associated with excessive necroptotic activity. In this way, new protocols can be developed to improve the management of high-risk pregnancies, including those at risk for preterm birth.

## Data Availability

All data generated or analyzed during this study are included in this published article, and all raw data will be provided by the corresponding author upon request.
